# Determinants of dietary behavior among youth: an umbrella review

**DOI:** 10.1186/s12966-015-0164-x

**Published:** 2015-02-01

**Authors:** Ester FC Sleddens, Willemieke Kroeze, Leonie FM Kohl, Laura M Bolten, Elizabeth Velema, Pam J Kaspers, Johannes Brug, Stef PJ Kremers

**Affiliations:** Department of Health Promotion, NUTRIM School for Nutrition and Translational Research in Metabolism, Maastricht University Medical Center+, PO Box 616, Maastricht, 6200 MD the Netherlands; Department of Health Sciences and the EMGO+ Institute for Health and Care Research, VU University Amsterdam, De Boelelaan 1085, 1081 HV Amsterdam, The Netherlands; Medical Library, VU University Amsterdam, PO Box 7057, 1007 MB Amsterdam, The Netherlands; Department of Epidemiology & Biostatistics and the EMGO+ Institute for Health and Care Research, VU University Medical Center, van der Boechorststraat 7, 1081 BT Amsterdam, the Netherlands

**Keywords:** Youth, Determinants, Dietary behavior, Umbrella review

## Abstract

**Background:**

The literature on determinants of dietary behavior among youth is extensive and unwieldy. We conducted an umbrella review or review-of-reviews to present a comprehensive overview of the current knowledge.

**Methods:**

Therefore, we included systematic reviews identified in four databases (i.e. PubMed, PsycINFO, The Cochrane Library and Web of Science) that summarized determinants of observable child and adolescent dietary behaviors. Data extraction included a judgment of the importance of determinants, strength of evidence and evaluation of the methodological quality of the eligible reviews.

**Results:**

In total, 17 reviews were considered eligible. Whereas social-cognitive determinants were addressed most intensively towards the end of the 20th century, environmental determinants (particularly social and physical environmental) have been studied most extensively during the past decade, thereby representing a paradigm shift.

With regard to environmental determinants, mixed findings were reported. Sedentary behavior and intention were found to be significant determinants of a wide range of dietary behaviors in most reviews with limited suggestive evidence due to the cross-sectional study designs. Other potential determinants such as automaticity, self-regulation and subjective norm have been studied in relatively few studies, but results are promising.

**Conclusion:**

The multitude of studies conducted on potential determinants of dietary behavior provides quite convincing evidence of the importance of several determinants (i.e. quite some variables were significantly related to dietary behavior). However, because of the often used weak research designs in the studies covered in the available reviews, the evidence for true determinants is suggestive at best.

## Background

Dietary behaviors have been found to track from childhood into adulthood [1]. Unhealthy food habits in childhood, therefore, can have a tremendous health impact later in life. Given the high prevalence of nutrition-related disease and mortality in Western countries [2], it is necessary to develop effective behavioral interventions to improve diet quality. But which factors determine a person’s dietary behavior? Interventions to improve health-related behaviors should be tailored to the most important and changeable determinants of these behaviors, preferably applying behavior change theories [3]. To facilitate improvement of relevant, effective programs and policies promoting healthy eating targeting dietary behavior it is important to identify the various factors that may influence children’s and adolescents’ food consumption.

**Figure 1:**
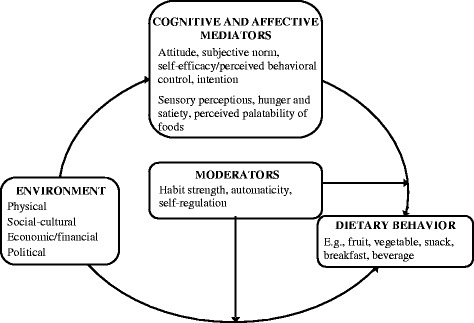
**Environmental research framework for weight gain prevention (EnRG), adapted from Kremers et al. [** 13 **].**

Socio-cognitive models of (health) behavior and behavior change, such as the Theory of Planned Behavior [4], Social-Cognitive Theory [5], and the Health Belief Model [6] have been applied frequently in development of nutrition education interventions. In very general terms -and not paying attention to the richness of and also differences between these models- these theories regard nutrition behavior to be determined by beliefs and conscious decisions, rational considerations of pros and cons of the behavior, perceived social influences, and assessment of personal efficacy and control. In additional fields of research, the physiological and affective influences on dietary behaviors have been studied, providing evidence for such basic factors as hunger and satiety, sensory perceptions, and perceived palatability of foods [7] as important drivers of food choice and dietary behaviors. And somewhat more recently, the so-called food environment that defines the availability and accessibility (i.e. physical environment), affordability (i.e. economic environment), social appropriateness or support (social-cultural environment), as well as rules, regulations and policies (i.e. political environment) regarding food choice and dietary behaviors has been studied in relation to food intake and dietary behaviors, as informed by (social) ecological behavior models [8-12]. Kremers and colleagues [13] proposed to integrate these insights in their Environmental Research framework for weight Gain prevention (EnRG; Figure 1). EnRG is a dual-process model and regards dietary behavior and physical (in) activity to be the result of direct ‘automatic’ responses to environmental cues (e.g. meal patterns and routines) as well as of more rational decision making based on cognitions such as intentions and beliefs. Furthermore, EnRG includes mediating pathways between environment and cognitions as well as potential moderators of the impact of these determinants such as habit strength and self-regulation skills.

The purpose of this study was to get a comprehensive and systematic overview of the scientific literature on correlates (referred to as potential determinants) and determinants of dietary behavior among children and adolescent (referred to as youth) to facilitate the improvement of effective healthy eating promoting interventions and identify gaps for future research initiatives. Because the scientific literature on this topic is unwieldy and has been documented in a number of systematic reviews in recent years, we aimed to conduct a review-of-reviews to provide a more comprehensive overview. We were interested in the association of all determinants that are potentially modifiable (social-cognitive, environmental, sensory and automatic processes) with observable dietary behavior (actual consumption behaviors like fruit consumption, beverage intake, snacking) among youth. By conducting a review-of-reviews, the so-called umbrella review, we aimed to (a) explore which determinant-behavior relationships have been studied so far, and (b) assess the importance and strength of evidence of potential determinants. The EnRG framework served to categorize the findings. Parallel to this umbrella review, a separate review-of-reviews of studies among adults was conducted by the same team with the same methodology [14]. Some parts of these two reviews -especially the description of the methodology- are therefore very similar.

## Methods

### Search strategy and eligibility criteria

To identify all relevant systematic reviews, we conducted systematic searches in the bibliographic databases PubMed, PsycINFO (via CSA Illumina), The Cochrane Library (via Wiley) and Web of Science for articles published between January 1, 1990 and May 1, 2014. The search terms included controlled terms, e.g. MeSH in PubMed, Thesaurus in PsycINFO, as well as free text terms (only in The Cochrane Library). Search terms expressing ‘food and dietary behavior’ were used in combination with search terms comprising ‘determinants’, ‘study design: (systematic) review’, ‘study population: humans’ and ‘time span (January 1, 1990 to May 1, 2014)’. The PubMed search strategy can be found in Table 1. The search strategies used in the other databases were based on the PubMed strategy.

**Table 1:** **Search strategy in PubMed: January 1st, 1990 to May 1st, 2014 (bottom-up): N = 13,156**

**Set**	**Search terms**
#6	#1 AND #2 AND #3 AND #4 NOT #5
#5	(“addresses” [Publication Type] OR “biography” [Publication Type] OR “case reports” [Publication Type] OR “comment” [Publication Type] OR “directory” [Publication Type] OR “editorial” [Publication Type] OR “festschrift” [Publication Type] OR “interview” [Publication Type] OR “lectures” [Publication Type] OR “legal cases” [Publication Type] OR “legislation” [Publication Type] OR “letter” [Publication Type] OR “news” [Publication Type] OR “newspaper article” [Publication Type] OR “patient education handout” [Publication Type] OR “popular works” [Publication Type] OR “congresses” [Publication Type] OR “consensus development conference” [Publication Type] OR “consensus development conference, nih” [Publication Type] OR “practice guideline” [Publication Type])
#4	“review” [tiab]
#3	“humans” [Mesh]
#2	(“food and beverages” [Mesh] OR “food” [tiab] OR “beverage” [tiab] OR “beverages” [tiab] OR “diet” [Mesh] OR “diet” [tiab] OR “eating” [Mesh] OR “eating” [tiab] OR “feeding behavior” [Mesh] OR “feeding behavior” [tiab] OR “feeding behaviour” [tiab] OR “drink” [tiab] OR “sodium chloride, dietary” [Mesh] OR “dietary sodium chloride” [tiab] OR “carbohydrates” [Mesh:noexp] OR “food habit” [tiab] OR “food habits” [tiab] OR “meal” [tiab] OR “meals” [tiab] OR “meal pattern” [tiab]) **NOT** “dietary supplements” [Mesh]) NOT “food additives” [Mesh]) NOT “micronutrients” [Mesh]) NOT “cannibalism” [Mesh]) NOT “carnivory” [Mesh]) NOT “herbivory” [Mesh]) NOT “bottle feeding” [Mesh]) NOT “breast feeding” [Mesh]) NOT “mastication” [Mesh])
#1	(“association” [Mesh] OR “association” [tiab] OR “associations” [tiab] OR “determinant” [tiab] OR “determinants” [tiab] OR “correlation” [tiab] OR “correlations” [tiab] OR “correlated” [tiab] OR “correlates” [tiab] OR “relation” [tiab] OR “relations” [tiab] OR “relationship” [tiab] OR “relationships” [tiab] OR “relate” [tiab] OR “related” [tiab] OR “relates” [tiab] OR “factor” [tiab] OR “factors” [tiab] OR “predict” [tiab] OR “predicted” [tiab] OR “prediction” [tiab] OR “predictive” [tiab] OR “predicts” [tiab] OR “predictor” [tiab] OR “associate” [tiab] OR “associates” [tiab] OR “associated” [tiab] OR “influence” [tiab] OR “influences” [tiab] OR “influencing” [tiab] OR “influenced” [tiab] OR “effect” [tiab] OR “effects” [tiab])

Note: Filters review; Publication data from 1990/01/01 to 2014/05/01; English.

Studies were included if they met the following criteria: (i) systematic reviews on observable food and dietary behavior (i.e. consumption behaviors like fruit intake and snacking consumption, not purchasing behavior); (ii) studies describing potential behavioral determinants; (iii) study design: (systematic) review; (iv) study population: humans and (v) time span: January 1, 1990 to May 1, 2014. We excluded: (i) studies that were not written in English; (ii) studies in which dietary behavior was not an outcome of the study; (iii); studies about dietary behaviors in disease management and treatment; (iv) studies that focused on specific population groups (e.g. chronically ill, pregnant women, cancer survivors); (v) studies not published as peer reviewed systematic reviews in scientific journals, e.g. theses, dissertations, book chapters, non-peer reviewed papers, conference proceedings, reviews of case studies and qualitative studies, design and position papers, umbrella reviews; (vi) reviews of studies on not directly observable dietary behavior (e.g. nutrient or energy intake, appetite); (vii) reviews of studies on non-modifiable determinants (e.g. physiological, neurological or genetic factors); (viii) reviews of studies on the effect of interventions (but reviews of experimental manipulation of single determinants were included); (ix) reviews not conducted systematically (search strategy including keywords and databases used not identified, and/or with too little information of the included studies presented). The current umbrella review focuses on youth (<18 years). A second umbrella review using the same methodology about determinants of dietary behavior in adults is published elsewhere [14].

### Selection process

**Figure 2:**
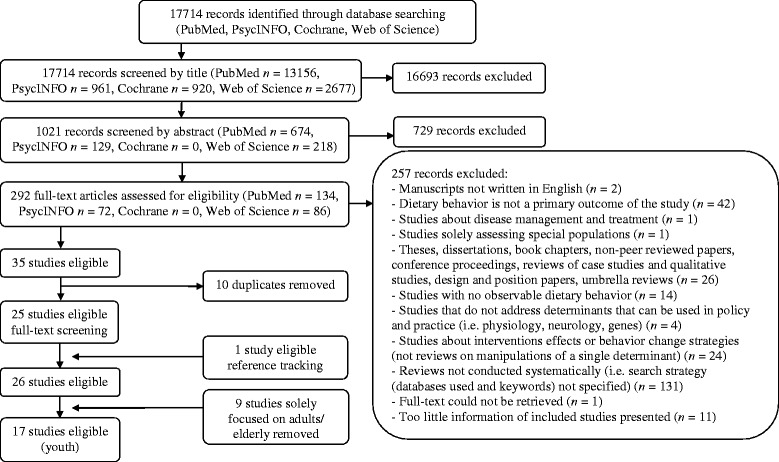
**Flow diagram of literature search by database.**

Figure 2 summarizes the manuscript selection process. In total, 17714 citations were obtained using PubMed (*n* = 13156), PsycINFO (*n* = 961), The Cochrane Library (*n* = 920), and Web of Science (*n* = 2677). The subsequent screening of the citations was performed by multiple reviewers (all citations were screened by ES and WK; some were screened by LB, SK, and EV). All titles of the citations were independently screened for relevance by two reviewers (ES and WK). Any disagreement was resolved by including the citation into the abstract screening process. Subsequently, abstracts of the remaining 1031 citations were retrieved for further screening. Another 729 citations were removed, resulting in 292 articles for full-text assessment for eligibility. In case of doubt, potential inclusion was discussed with a third reviewer (SK). Studies that did not meet the inclusion criteria (*n* = 257) were removed. Figure 2 displays the reasons for exclusion. Additionally, duplicates (*n* = 10) were removed. Thereafter, the reference lists of all review papers selected for inclusion (*n* = 25) were scanned for further relevant references. This reference tracking technique resulted in one additional review article appropriate for inclusion. In total, 26 reviews were considered eligible. However, of these reviews, 9 were only focused on determinants of adult dietary behavior (references reported in the umbrella review about determinants of dietary behavior in adults of Sleddens et al. [14]). Five reviews assessed dietary behavior of both youth and adults [15-19]. Therefore, 17 reviews were considered eligible for our umbrella review on determinants of youth dietary behavior [15-31].

### Data extraction including rating of methodological quality

Four authors (ES, WK, LK, and LB) extracted data from the selected reviews. The extracted data included search range applied, total number of studies included in the reviews and number of studies included in the reviews that are eligible for the current umbrella review, total number of participants of included studies in the reviews and number of participants of the included studies that are eligible for the current umbrella review, and age and continent of included eligible studies. For a description of the results, correlate and outcome measures were extracted, as well as overall results of the reviews and overall limitations and recommendations of reviews. Additionally, the methodological quality of the reviews was evaluated using quality criteria adapted from De Vet, De Ridder, & De Wit [32] and based on the Quality Assessment Tool for Reviews [33]. In total, a review was scored on eight criteria (with a total quality scoring ranging from 0–8) (see Table 2); 0 when the criteria was not applicable for the included review; 1 when the criteria was applicable for the included review. Disagreement between the reviewers on individual items were identified and solved during a consensus meeting. The quality of the reviews could be labeled as weak (quality scores ranging from 0 to 3), moderate (quality scores ranging from 4 to 6) or strong (quality scores ranging from 7 to 8). Furthermore, we judged the importance of included determinants in the reviews and judged its strength of evidence. The importance of a determinant refers to the statistical significance of a potential determinant and/or effect size estimate in relation to a particular type of dietary behavior. It refers to the amount of reviews (or eligible studies within the reviews) that did or did not find statistically significant results. For a particular determinant to receive the highest ranking (highest level of importance), all eligible studies in each review should have found a significant relationship and/or reported a (non)-significant effect size larger than 0.30. The strength of evidence represents the consistency between study findings and designs of the studies. Longitudinal observational studies and -where relevant- experimental studies of sufficient size, duration and quality showing consistent effects were given prominence as the highest ranking study designs. For this judgment we applied two coding schemes, see Tables 3 and 4 respectively. The criteria for grading evidence were adapted from those of the World Cancer Research Fund [34]. The data extraction method is similar to the study of Sleddens et al. [14].

**Table 2:** **Quality assessment of reviews of determinants of dietary behavior among youth**

**Quality assessment criteria**	**Was there a clearly defined search strategy?** ^**1**^	**Was the search strategy comprehensive?** ^**2**^	**Are inclusion/ exclusion criteria clearly stated?**	**Are the designs and number of included studies clearly stated?**	**Has the quality of primary studies been assessed?**	**Did the quality assessment include study design, study sample, outcome measures or follow-up (at least 2 of 4)**	**Does the review integrate findings beyond describing or listing findings of primary studies?**	**Has more than one author been involved in the data screening and/or abstraction process?**	**Sum quality score**
Williams [31]	1	1	1	1	1	1	1	1	8
Gardner [17]*	1	1	1	1	1	1	1	0	7
Pearson & Biddle [19]*	0	1	1	1	1	1	1	0	6
Adriaanse [15]*	0	1	1	1	0	0	1	1	5
McClain [24]	0	1	1	1	1	1	1	1	7
Van der Horst [29]	0	1	1	1	0	0	1	1	5
Pearson [25]	1	1	1	1	0	0	1	0	5
De Craemer [21]	1	1	1	1	0	0	1	1	6
Pearson [26]	1	1	1	1	1	1	1	0	7
Verloigne [30]	1	1	1	1	1	1	1	0	7
Ford [22]	1	1	1	1	1	1	1	1	8
Caspi [16]*	0	0	1	0	0	0	1	0	2
Moore & Cunningham [18]*	1	1	1	1	0	0	1	1	6
Lawman & Wilson [23]	0	1	1	1	1	1	1	0	6
Sleddens [28]	0	1	1	1	0	0	1	1	5
Berge [20]	0	0	1	1	1	1	1	0	5
Rasmussen [27]	0	1	1	1	1	1	1	1	7
	8/17	15/17	17/17	16/17	10/17	10/17	17/17	9/17	

*Note*: ^1^A search is rated clearly defined if at least search words and a flow chart is presented; ^2^A search is rated as comprehensive if at least two databases and the reference lists of examined papers were searched. *Reviews also including adults; weak (score ranging from 0–3) n = 1 (5.88%), moderate (score ranging from 4–6) n = 9 (52.94%), strong (score ranging from 7–8) n = 7 (41.18%).

**Table 3:** **Importance of a determinant**

**Five categories of importance are defined: ++ / + / 0 / - / --**	
**The categories are defined as follows:**	
++	The variable has been found to be a statistically significant determinant in all identified reviews, without exception. This could mean that only one review has included a particular variable, and showed that this was a significant correlate and/or reported a (non)-significant effect size larger than 0.30, but it could also mean that a number of reviews were conducted that included this variable and all of them concluded that the variable was significantly related to the particular behavioral outcome.
+	The variable has been found to be a statistically significant determinant and/or reported a (non)-significant effect size larger than 0.30 in most reviews or studies within the review, with some exceptions. This implies that > 75% of the available reviews concluded the variable to be related, or the separate reviews report that 75% or more of the original studies concluded the factor to be related. This could therefore mean that only one review has included a particular variable, and showed that this was a significant correlate in > 75% of studies. But it could also mean that a number of reviews were executed towards this variable and most, but not all, concluded that the variable was significantly related to the particular behavioral outcome.
0	The variable has been found to be a determinant and/or reported a (non)-significant effect size larger than 0.30 in some reviews (25% to 75% of available reviews or of the studies reviewed in these reviews), but not in others. This could mean that only one review has included a particular variable, and showed ‘mixed findings’, but it could also mean that results are mixed across reviews.
-	The variable has been found not to be a determinant, with some exceptions. This implies that <25% of the available reviews or of the original studies in the included reviews concluded that the variable was related. This could thus mean that only one review has included a particular variable, and generally showed ‘null findings’, with some exceptions. But it could also mean that a number of reviews were executed towards this variable and most, but not all, concluded that the variable was not significantly related to the particular behavioral outcome.
--	The variable has been found not to be related to this particular outcome. The absence of a relation was identified in all identified reviews, without exception. This could mean that only one review has included a particular variable, and showed that this correlate was not related to the behavior in question, but it could also mean that a number of reviews were executed towards this variable and all of them concluded that the variable was unrelated to the particular behavioral outcome.

**Table 4:** **Criteria for grading evidence, see World Cancer Research Fund** [34] **for the full list**

**Strength of evidence:**	
Ideally the definition of the strength of evidence should be based on a relationship that has been established by multiple randomized controlled trials of manipulations of single isolated variables, but this type of evidence is often not available.	
The following criteria were used to describe the strength of evidence in this report. They are based on the criteria used by the World Cancer Research Fund (World Cancer Research Fund, 2007 [34]), but have been modified for the research question at hand. Four categories were defined: convincing/probable/limited, suggestive/limited, no conclusion.	
Convincing evidence:	Evidence based on studies of determinants showing consistent associations between the variable and the behavioral outcome. The available evidence is based on a substantial number of studies including longitudinal observational studies and where relevant, experimental studies of sufficient size, duration and quality showing consistent effects. Specifically, the grading criteria include evidence from more than one study type and evidence from at least two independent cohort studies should be available, and strong and plausible experimental evidence.
Probable evidence:	Evidence based on studies of determinants showing fairly consistent associations between the variable and the behavioral outcome, but there are shortcomings in the available evidence or some evidence to the contrary, which precludes a more definite judgment. Shortcomings in the evidence may be any of the following: insufficient duration of studies, insufficient studies available (but evidence from at least two independent cohort studies or five case-control studies should be available), inadequate sample sizes, incomplete follow-up.
Limited, suggestive evidence:	Evidence based mainly on findings from cross-sectional studies. Insufficient longitudinal observational studies or experimental studies are available or results are inconsistent. More well-designed studies of determinants are required to support the tentative associations.
Limited, no conclusive evidence:	Evidence based on findings of a few studies which are suggestive, but are insufficient to establish an association between the variable and the behavioral outcome. No evidence is available from longitudinal observational or experimental studies. More well-designed studies of determinants are required to support the tentative associations.

## Results

### Description of reviews

Quality assessment ratings are presented in Table 2. One review received a quality rating of 2 (weak). The other reviews were rated as moderate (n = 9) or strong (n = 7). In all reviews, the inclusion and exclusion criteria were clearly stated and the review did integrate findings beyond describing or listing findings of primary studies. Clearly defined search strategies were absent in more than half of the reviews (9 out of 17 reviews), as usually a flow chart of the data screening process was missing.

Table 5 provides an overview of the characteristics of the included reviews. In three reviews [22-24] all included studies were eligible for our current study. Most of the studies included in the reviews used a cross-sectional study design. Six studies did not provide any information about sample sizes [15,16,19,21,24,29]. The remaining reviews included a total sample size of 695 to 570,403. The target groups of the eligible studies ranged in ages between the different reviews, although the focus was on primary school-aged children and adolescents. Most of the studies included in the reviews were conducted in North-America, followed by Europe.

**Table 5:** **Characteristics of analyzed systematic reviews among youth**

**Author and date**	**Search range applied**	**Number of eligible studies included in the review/total number of studies included in the review**	**Designs of studies**	**Total sample size of eligible studies included in the review/Total sample size of all studies included in the review**	**Ages**	**Continent**
Williams et al., 2014 [31]	Up to 2013	13/30	Cross-sectional n = 12, longitudinal n = 1	Total n = 570,403, range 610 to 529,367/Total n = 1,550,415 (26 studies, NR: 4 studies), range 319 to 926,018	11 to 17y	North-America n = 6, Europe n = 5, Australasia n = 1, Asia n = 1
Gardner et al., 2011 [17]	No date limits were set.	4 studies (2 samples)/22 studies (21 samples)	Cross-sectional n = 4	Total n = 695, range 312 to 383/Total n = 6,121, range 93 to 876	High school students	Only Europe
Pearson & Biddle, 2011 [19]	NR	31 studies (240 samples (total), 33 samples (children), 207 samples (adolescents))/53 studies (111 samples)	Majority cross-sectional	NR/Children: mean n = 1,184, range 66 to 6,235, Adolescents: mean n = 8,356, range 60 to 14,407	<12, 12 to 18y	Children: majority North-America n = 13, Adolescents: half North-America n = 13
Adriaanse et al., 2011 [15]	Up to 2009	1 study/21 research articles describing 23 empirical studies	Healthy eating: cross-sectional n = 1, prospective n = 3, interventions n = 11; Unhealthy eating: longitudinal n = 1, interventions n = 8	NR/NR	11 to 16y	NR
McClain et al., 2009 [24]	From 1990 to 2009	50 studies/77 studies	Overall results: cross-sectional n = 64, prospective n = 11, interventions n = 2	NR/<50 n = 4, 51–99 n = 1, 100–499 n = 39, 500–999 n = 15, 1000–2999 n = 14, 3000–4999 n = 3, ≥5000 n = 3	<13, 13 to 18y	Overall results: North-America n = 48, Europe n = 32, Australasia n = 3, Africa n = 3, Asia n = 2, other n = 3
Van der Horst et al., 2007 [29]	From 1980 to 2004	36 studies (44 samples)/58 studies (77 samples)	Cross-sectional 95%, longitudinal 3%, case control 2%	NR/<100 n = 8, 100–199 n = 6, 200–299 n = 1, 300–499 n = 5, 500–999 n = 2, 1000–2999 n = 9; 3000–4999 n = 4, ≥5000 n = 2	<13, 13 to 18y	North-America 45 samples, Europe 26 samples, Asia 4 samples, Oceania 2 samples
Pearson et al., 2009 [25]	Up to 2008	24 studies (33 samples)/ 24 studies (33 samples)	Majority cross-sectional n = 23	Children: mean n = 1,534, range 136 to 4,314. Adolescents: mean n = 2,533, range 357 to 18,177	6 to 18y	Majority Europe n = 12 (papers)
De Craemer et al., 2012 [21]	From 1990 to 2010	6 studies/43 studies	Cross-sectional n = 35, longitudinal n = 6, cross-sectional and longitudinal n = 1, intervention n = 1	NR/<100 n = 3, 100–999 n = 28, >1000 n = 12. Study sample sizes ranged from 46 to 5,652	4 to 6y	North-America n = 21, Europe n = 9, Australasia n = 12, Asia n = 1
Pearson et al., 2009 [26]	Up to 2007	Total papers: n = 60. Children: 25 studies (33 samples). Adolescents: 38 studies (55 samples)/Total papers: n = 60. Children: 25 studies (33 samples). Adolescents: 38 studies (55 samples)	Majority cross-sectional n = 24. Children: cross-sectional n = 31, longitudinal n = 2; Adolescent: cross-sectional n = 55 , longitudinal n = 0	Mean n = 1,131, range 536 to 8,263. Children (6-11y): < 100 n = 8, 100–199 n = 2, 200–299 n = 1, 300–499 n = 1, 500–999 n = 6, 1000–2999 n = 10, 3000–4999 n = 3, unknown n = 2; Adolescent (12-18y): < 100 n = 2, 100–199 n = 3, 200–299 n = 3, 300–499 n = 11, 500–999 n = 4, 1000–2999 n = 12, 3000–4999 n = 8, >5000 n = 10	6 to 18y Children: 6-11y Adolescents: 12-18y	Children: North-America n = 15, Europe n = 15, Australasia n = 1, South-America n = 1, Asia n = 2; Adolescent: North- America n = 23, Europe n = 16, Australasia n = 9, South-America n = 2, Asia n = 5
Verloigne et al., 2012 [30]	From 1990 to 2010	17 studies/76 studies	Cross-sectional n = 16, longitudinal n = 1	100-199 n = 1, 300–499 n = 1, 500–999 n = 5, 1000–2999 n = 5, 3000–4999 n = 2, ≥5000 n = 3/< 100 n = 3, 100–199 n = 9, 200–299 n = 6, 300–499 n = 10, 500–999 n = 17, 1000–2999 n = 20, 3000–4999 n = 5, ≥5000 n = 6	10 to 12y	North-America n = 9, Europe n = 4, Australasia n = 4
Ford et al., 2012 [22]	NR	9 studies /12 studies	Cross-sectional n = 9	Total n = 13,280, range 240 to 4,983/Total n = 13,386, range 106 to 4,983	2 to 6y	NR
Caspi et al., 2012 [16]	Up to 2011	5 studies /38 studies	Majority cross-sectional, intervention n = 3	NR/NR	Children5 to 6y, 10 to 12y, youth, boy scouts	North-America n = 4, Australasia n = 1
Moore & Cunningham, 2012 [18]	NR	2 studies /14 studies	Cross-sectional n = 2	Total n = 5,144, range 824 to 4,320/Total n = 94,230, range 51 to 64,277	Preteens and 14 to 17y	NR
Lawman & Wilson, 2012 [23]	From 1995 to 2010	11 studies /38 studies	Cross-sectional n = 8, longitudinal n = 3.	Total n = 21,865, range 228 to 4,746/Total n = 51,396, range 52 to 7,907	9 to 21y	North-America n = 32
Sleddens et al., 2011 [28]	Up to 2010	10 studies /36 studies	Cross-sectional: n = 9, longitudinal: n = 1	Total n = 14,567, range 74 to 4,555/Total n = 35,146, range 48 to 4,983	NR	North-America n = 5, Europe n = 5
Berge, 2009 [20]	From 2000 onwards	48 studies /81 studies	Cross-sectional n = 39, longitudinal n = 8, intervention n = 11	Total n = 190,270, range 23 to 99,426/Total n = 276,557, range 23 to 99,426	0 to 18y	NR
Rasmussen et al., 2006 [27]	Up to 2005	98 studies /98 studies	Cross-sectional n = 90, longitudinal n = 8	<500 n = 24, 500–1000 n = 20, >1000 n = 53, NR: n = 1/<500 n = 24, 500–1000 n = 20, >1000 n = 53, NR: n = 1	NR	North-America n = 50, Europe n = 31, Australasia n = 16, South-America n = 1

*Note*: Designs of studies: cross-sectional, longitudinal observational, case control, and intervention studies (experimental, behavioral laboratory, filed studies in which interventions were studied); NR: not reported; we were mainly interested to provide a more thorough description on the eligible studies of the included reviews (designs of studies, ages, continent).

### Findings of the reviews

Table 6 provides an overview of the correlates and outcomes (i.e. observable dietary behaviors) included in the reviews, and the overall findings, limitations and recommendations reported by the authors of these reviews. In the following two paragraphs we give an overview of the determinant-behavior relationships that have been studied so far, and give an overview of the importance and strength of evidence of potential determinants.

**Table 6:** **Results of the reviews about determinants of dietary behavior among youth**

**Author, date**	**Outcome measures**	**Correlate measures**	**Overall results of the reviews**	**Overall limitations of the review**	**Overall recommendations of the review**
Williams et al., 2014 [31]	Sugar-sweetened beverages, fast food, fruit and vegetables	Food and retail outlets	Little evidence for an association between retail food environment surrounding schools and food consumption.	1) Meta-analysis not possible due to different conceptualizations and measures of the food environment surrounding schools.	1) Longitudinal studies needed.
					2) Integrate validated classification systems of the retail food environment, explore the capacity of alternative methods for validating exposure data.
				2) Loss of detail; a review is dependent upon outcomes and analyses that individual papers reported.	
					3) Specify individual-level measures of exposure to the food environment.
					4) Collecting complementary measures of both qualitative and quantitative measures of food access.
					5) Collect outcome measures that are appropriate relative to the exposures.
					6) Take age and ethnicity differences into account.
Gardner et al., 2011 [17]	Sugar-sweetened soft drinks	Habit strength	The weighted habit–behavior correlation effect estimate for nutritional habits was moderate to strong in size (fixed: r + =0.43; random: r + =0.41), and effects were of equal magnitude across healthful (fixed: r + =0.43; random: r + = 0.42) and unhealthful (fixed: r + =0.42; random: r + =0.41) dietary habits. The medium-to-large grand weighted mean habit–behavior correlation (r + ≈0.45) suggests that habit alone can explain around 20% of variation in nutrition related behaviors (i.e. R2 ≈ 0.20).	1) While it was not possible to meta-analyze interaction effects, habit often moderated the relationship between intention and behavior, such that intentions had reduced impact on behavior where habit was strong. This finding must be interpreted cautiously as it may reflect a bias towards publication of studies which find significant interaction, and so an overestimation of the robustness of this effect.	1) Explorations of the role of counter-intentional habits on the intention–behavior relationship, such as the capacity for habitual snacking to obstruct intentions to eat a healthful diet, are needed.
					2) Healthful behaviors can habituate. The formation of healthful (‘good’) habits, so as to aid maintenance of behavior change, thus represents a realistic goal for health promotion campaigns.
					3) More methodologically rigorous research is required to provide more conceptually coherent and less biased observations of the influence of habit on action.
				2) Many studies were cross-sectional, and so modeled habit as a predictor of past behavior. This fails to acknowledge the expected temporal sequence between habit and behavior, and is also conceptually problematic given that, at least in early stages of habit formation, repeated action strengthens habit.	4) A more comprehensive understanding of nutrition behaviors, and how they might be changed, will be achieved by integrating habitual responses to contextual cues into theoretical accounts of behavior.
				3) Reliance on self-reports of behavior.	
Pearson & Biddle, 2011 [19]	Fruit, vegetables, fruit and vegetable intake combined, energy-dense snacks, fast foods, energy-dense drinks	Sedentary behavior: screen time (TV viewing, video/DVD, computer use, sitting while talking/reading/homework)	Sedentary behavior, usually assessed as screen time and predominantly TV viewing, is associated with unhealthy dietary behaviors in children and adolescents. There appears no clear pattern for age acting as a moderator. There appears to be more consistent associations between sedentary behavior and diets for women/girls than for men/boys.	1) Many studies were cross-sectional.	1) More studies using objective measures of sedentary behaviors and more valid and reliable measures of dietary intake are required.
				2) Use of self-report measures of sedentary and dietary behaviors that lack strong validity.	
					2) Examine the longitudinal association between sedentary behavior and dietary intake, and the tracking of the clustering of specifıc sedentary behaviors and specifıc dietary behaviors. For example, it appears from the mainly cross-sectional evidence presented that TV viewing is associated with unhealthy dietary patterns. Much less is known about diet and either computer use or sedentary motorized transport. It is likely that the main associations will be with TV, but this needs testing.
				3) Sedentary behavior is largely operationally defined as screen time, and this is mainly TV viewing, making it diffıcult to draw any conclusions regarding non-screen time and dietary intake.	
				4) Although “screen time” can include TV and computer use, this does not help in identifying whether it is TV, computer use, or both, that is associated with unhealthy diets.	
					3) A focus on sedentary behaviors and dietary behaviors that “share” determinants as well as determinants of the clustering of sedentary and dietary behaviors will aid the development of targeted interventions to reduce sedentary behaviors and promote healthy eating.
Adriaanse et al., 2011 [15]	Fruit and vegetable consumption	Use of implementation intentions	Considerable support was found for the notion that implementation intentions can be effective in increasing healthy eating behaviors, with twelve studies showing an overall medium effect size of implementation intentions on increasing fruit and vegetable intake. However, when aiming to diminish unhealthy eating patterns by means of implementation intentions, the evidence is less convincing, with fewer studies reporting positive effects, and an overall effect size that is small.	NR	1) Although implementation intention instructions were not included as a moderator in the present meta-analysis due to the limited amount of studies, it seems prudent that future research takes into account the importance of using autonomy supportive instructions.
					2) Stricter control conditions as well as better outcome measures are required.
					3) Investigate efficacy of implementation intentions in diminishing unhealthy eating behaviors. In doing so, these studies should also compare the efficacy of different types of implementation intentions, as these may have differential effects on unhealthy food consumption.
McClain et al., 2009 [24]	Fruit, juice and vegetable consumption, sugar, snacking, sweetened beverage consumption	Psychosocial correlates like: attitude, availability, intention, knowledge, norms, self-efficacy, preferences, parental factors, and more.	Perceived modeling and dietary intentions to make healthy or less healthy dietary changes (such as intentions to decrease consumption of sugary beverages or intentions to increase consumption of medium fat milk) have the most consistent and positive associations with dietary behavior. Other psychosocial correlates such as liking, norms, and preferences were also consistently and positively associated with dietary behavior in children and adolescents. Availability, knowledge, outcome expectations, self-efficacy and social support did not show consistent relationships across dietary outcomes.	1) Many studies were cross-sectional.	1) Future intervention research may benefit from the incorporation of findings from this review to create more effective adolescent and childhood dietary interventions by targeting the variables shown in this review that are most consistently associated with the various eating behaviors such as intentions, modeling, norms, liking, and preferences.
				2) Not possible to conduct meta-analysis.	
				3) Authors combined conceptually similar psychosocial determinants into one category, which may have introduced bias.	
				4) Most studies relied on self-report of dietary intake.	2) Investigate variables that have been insufficiently examined to date, particularly the variables rooted in affective theories. It is quite plausible that affective factors, such as motivation, executive control, or meanings of behavior might drive the dietary behavior of children and adolescents.
				5) Bias might have been introduced due to possible lack of validity or reliability of both dietary and psychosocial measures.	
				6) Certain studies reported only significant findings and did not address non-significant findings.	3) Investigate psychosocial correlates of several dietary behaviors that are known to influence weight and metabolic health such as fat and fiber that have been understudied.
				7) Only studies included that were published in English in peer-reviewed journals (electronic databases).	
				8) This review did not separate children and adolescents into distinct categories, although research has suggested that children and adolescents exhibit different health behaviors.	
Van der Horst et al., 2007 [29]	Fruit intake, vegetable intake, juice intake, composite measure of fruit and vegetable intake, composite measure of fruit juice and vegetable intake, fast food consumption, snack food intake, pizza and snack, soft drink consumption.	Correlates are categorized under home/household, educational institutions, neighborhood, city/municipality.	Consistent evidence, for the relationship between parental intake and children’s fruit and vegetable intake, and for parent educational level with adolescent’s fruit and vegetable intake. A positive association was found for the relationship between availability and accessibility with children’s fruit and vegetable intake. Further positive associations were found for modeling (fruit/vegetable), parental intake (soft drink), parenting style (fruit/vegetable), family connectedness (fruit/vegetable) and encouragement to increase food intake (fruit/vegetable).	1) Many potential environmental determinants have been examined for a variety of dietary behaviors, but only few studies have been conducted on the same specific environmental factor—dietary behavior combination.	1) Replication of studies on the same specific environmental factors is necessary, to generate more compelling evidence for associations between environmental factors and dietary intake.
					2) The finding that parental behavior is associated with child and adolescent intakes implies that interventions should take the behavior of parents into account, or desensitize adolescents for the (unfavorable) behavior of their parents. Parents should be more strongly encouraged to give the right example, especially where fat and energy intakes are concerned.
				2) Many studies were cross-sectional.	
				3) Reliance on self-report measures.	
				4) Similar environmental determinants were collapsed conceptually into one category, although potential determinants in the same category were often dissimilar or measured in different ways.	
					3) Fruit and vegetable promotion should focus especially on adolescents from parents with lower levels of education.
				5) Only studies included that were published in English in peer-reviewed journals (electronic databases).	4) Studies are needed that target the environmental levels and factors that have found to be (nearly) empty in the ANGELO framework, such as physical, socio-cultural, economic and political factors in the school (e.g. school food policy and food prices), neighborhood (e.g. availability and accessibility of foods in shops) and city/ municipality environment (e.g. food policy, food prices, marketing). Factors such as availability and accessibility at home, school and neighborhood should be studied in relation to energy, fat, soft drink, snacks and fast food intake.
				6) Studies were heterogeneous in the conceptualization, measurement of the environmental determinant and/or dietary intakes, samples and analyses used: not possible to assess the overall strength of associations.	
				7) Multiple environmental factors examined in one study were included in the review, so these associations are not independent.	
					5) Need for longitudinal studies with valid or objective measures.
Pearson et al., 2009 [25]	Breakfast consumption, breakfast skipping	Physical (availability and accessibility), food poverty, socio-cultural (e.g. two parent family, modeling, family communication, monitoring, food rules, parental presence), demographic (SES, parental education level and employment)	This review reported support for three family variables: Parental breakfast eating and living in two parent families were positively associated with adolescent breakfast consumption; and socio-economic deprivation was inversely associated with breakfast consumption.	1) Several studies may not have been powered to detect significant associations between family correlates and breakfast behaviors.	1) Future studies should clearly define breakfast foods (e.g. breakfast cereal, breads, milk, snacks on the run) being measured as this will allow for an understanding of the healthfulness of this behavior and will provide scope for interventions to promote healthy breakfast consumption.
				2) Diversity in the definition of breakfast across the literature.	
					2) Importance of family structure should be considered when designing programs to promote breakfast consumption.
					3) Future qualitative studies are needed to further explicate the mechanisms of the complex relationship between SES and adolescent breakfast behaviors.
De Craemer et al., 2012 [21]	Sweet beverages, fruit and vegetable intake combined, snacks, milk intake	Demographic and biological variables, behavioral variables, physical environmental variables	TV viewing was positively associated with the intake of sweet beverages, snacks and inversely associated with fruit and vegetable intake. Parental modeling was associated with fruit and vegetable intake. No association with fruit and vegetable intake was found for restriction of eating, and an indeterminate result was found for pressuring the child to eat. Food availability was not associated with fruit and vegetable intake and snacking but had an indeterminate result for sweet beverages.	NR	1) Future research should investigate similar correlates of physical activity, sedentary behavior and eating behavior to develop more efficient interventions.
					2) Future research should be on interventions to predict whether interventions targeting these correlates will have an impact.
					3) Future research should focus on identifying the common correlates of physical activity, sedentary behavior and eating behavior in preschool-aged children so that better tailored interventions could be developed.
					4) More longitudinal studies are needed.
Pearson et al., 2009 [26]	Fruit and vegetable consumption separately. fruit, fruit juice and vegetable consumption combined.	Physical (e.g. availability, accessibility), socio-cultural (parental modeling, parental intake, family rules), and demographic correlates (e.g. SES).	Children: home availability, family rules (demand/allow) and parental encouragement were positively associated with children’s fruit and vegetable intake. Parental modeling and parental intake were positively associated with children’s consumption of fruit and fruit juice and vegetable intake. Adolescents: parental intake and parental occupational status were found to be positively associated with adolescents’ consumption of fruit. Parental intake was also positively associated with adolescents’ vegetable consumption. There is also evidence for a positive association between parental education and adolescents’ fruit juice and vegetable intake.	1) Diversity in character (e.g. measures used and correlates studied)	1) More longitudinal studies are needed.
					2) More studies are needed to test understudied correlates to generate more convincing evidence for associations between correlates and dietary behaviors. 3) Studies should report the validity and reliability of measures used to assess predictor variables.
				2) Difficult to assess overall consistency of associations.	
				3) Several studies may not have been powered to detect significant associations between family correlates and dietary behaviors.	
				4) Few studies have examined the same specific combination of family correlate and dietary behavior, thus limiting the possibilities of drawing strong or consistent conclusions.	
				5) Many studies were cross-sectional.	
				6) Reliance on self-report measures.	
				7) Little data on reliability and validity of measures of dietary outcomes and physical and socio-cultural family correlates.	
				8) Only studies included that were published in English	
Verloigne et al., 2012 [30]	Breakfast consumption, soft drink consumption.	Family and school environment: physical, socio-cultural, economic, political correlates.	Parental descriptive/ injunctive norms and control/supervision were positively related to breakfast. Parental catering on demands, avoidance of negative modeling behavior, permissiveness, and area deprivation were inversely related. School SES was negatively related and teacher injunctive norms was positively related to breakfast. Availability at home, parental soft drink, and permissive parenting style were positively related to soft drink. Having family dinners, household income, parental employment status, and limits were inversely related. Availability of soft drinks at school and intake at school were positively related with soft drink. Participation in healthy school lunches was inversely related.	1) Only studies included that were published in English	1) More longitudinal studies are needed.
					2) Interventions could help parents to create a supportive environment for their children to promote healthy behavior.
				2) Did not take possible moderators and covariates into account.	
					3) More research is needed to focus on important school-environmental factors when developing an intervention program.
				3) Not all existing studies on this topic were covered.	
				4) Focused on the consistency of the association and not on the strength of the association.	
				5) Conceptually similar variables were combined into a single category, even if variables were measured in a different way.	
				6) Many studies were cross-sectional.	
Ford et al., 2012 [22]	Vegetables, fruit and vegetable intake, (non)-fruit juice, high-energy/sugar-sweetened drinks, whole or 2% milk, fast foods, breakfast	TV, video, and computer time in minutes.	Eleven of the 12 included studies reported significant associations between TV and adverse dietary behaviors in young children. Six studies reported significant inverse relationships between TV viewing and fruit and vegetable intake.	1) Reliance on parent-reported methods to assess child TV viewing.	1) Guidelines for TV viewing use in young children should be further delimited.
				2) Many studies were cross-sectional.	2) More longitudinal studies are needed.
					3) Direct measurement of TV use.
Caspi et al., 2012 [16]	Fruit and/or vegetables intake, 100% fruit juice consumption.	Food environment: 5 dimensions of food access (availability, accessibility, affordability, accommodation, acceptability).	Moderate evidence in support of the causal hypothesis that neighborhood food environments influence dietary health. Perceived measures of availability were consistently related to multiple healthy dietary outcomes.	NR	1) More standardized/validated measures for food environment assessment needed.
					2) Develop/refine understudied measures.
					3) Abandon purely distance-based measures of accessibility, and combine multiple environmental assessment techniques.
					4) Researchers should continue to expound upon the conceptual definitions of food access as they develop and refine new combinations of measure for the food environment.
Moore & Cunningham, 2012 [18]	Daily fruit and vegetable consumption, snacking, breakfast consumption, soda consumption, meat intake.	Social status, stress.	Higher stress is related to less healthy dietary behaviors. The majority of studies reported that higher social position is related to healthier diet.	1) Only studies included that were published in English.	1) More quantitative dietary assessment tools such as FFQ, repeated 24-hr recalls, and food diaries are needed.
				2) Many studies were cross-sectional.	2) More longitudinal studies are needed.
				3) Because obesity results from a prolonged period of positive energy imbalance, assessment of dietary behaviors at a single point in time makes inferences related to diet and obesity difficult.	3) Important to acknowledge additional factors that influence energy intake, such as SES and stress levels.
					4) Implementing appropriate monitoring and evaluation is essential to identifying successful, holistic strategies that can be used to improve quality of care.
				4) Heterogeneity of measures.	
Lawman & Wilson, 2012 [23]	Fruit, vegetables, fast food, soft drink, dairy, milk, breakfast.	Parenting (parental support for health behaviors, parenting style and parental monitoring surrounding health behaviors) and/or environmental factors (home availability/access, neighborhood availability/access and the built environment, neighborhood safety, neighborhood social factors).	The current review found support for some parenting and physical environmental factors for health behaviors, particularly parental monitoring and neighborhood social factors.	1) Many studies were cross-sectional.	1) More longitudinal studies are needed on at-risk youth.
				2) Reliance on self-report measures.	2) More objective measures of health behaviors or multiple reporters, who may hold different perspectives are needed, when objective measures are not feasible.
					3) Future research should be conscious of reporting results in a way that facilitates systematic review of the literature
					4) Examine additional levels of the bio-ecological model such as interpersonal and other macro- or society and policy level factors.
					5) Future research should explore the relation between home/environment and health behaviors, particularly neighborhood social contextual factors such as social cohesion, and how factors at multiple bio-ecological levels may be influencing them (e.g. moderators).
					6) More research monitoring is needed.
					7) Development of more valid measures of parenting, family, and home environment variables is warranted.
					8) Examine how parenting style is related to health behavior outcomes.
Sleddens et al., 2011 [28]	Vegetables, fruit, sugar-sweetened beverages, soft drinks, breakfast, snacks/sweets	General parenting	In many studies significant associations with general parenting were found. Generally, children raised in authoritative homes were found to eat healthier.	1) Reliance on questionnaires and parental self-report measures.	1) Additional research is needed to further study the influence of mediating and moderating factors influencing the general parenting - child weight relationship, preferably employing a longitudinal design with more extended follow-up periods.
				2) Differences in conceptualization of parenting constructs across studies.	
				3) Different categorizations to classify parents into styles across studies.	2) More longitudinal studies are needed using diverse ethnic samples and age groups.
				4) Heterogeneity of measurements across studies and lacking information about distribution of independent and outcome variables.	3) Larger samples of fathers should be included to allow for comparisons between mothers and fathers.
				5) Few studies examined the role of general parenting as a contextual factor that can influence the effectiveness of food-related parenting practices in predicting children’s dietary intake behaviors (moderation analyses).	4) Intervention developers should increase their attention to the family context as it is an important factor influencing outcomes of overweight interventions for children.
Berge, 2009 [20]	Fruit and vegetable consumption, sugar sweetened beverages, dairy products, breakfast consumption, etc.	Parental domain (e.g. parenting style, parenting practices), family functioning domain (e.g. family meals, family emotional closeness/ connection, family weight teasing).	Parental domain: authoritative parenting style is positively associated with dietary intake. Family functioning domain: from cross-sectional and longitudinal research there is convincing evidence that family meals have an enduring protective factor for children and adolescents, girls and boys, and across diverse ethnic groups related to healthy dietary intake.	1) Many studies were cross-sectional.	1) More longitudinal, experimental and direct observational research in all family domains is needed.
				2) Many studies used single-group designs.	
				3) Reliance on self-report measures.	2) Beneficial to incorporate mixed qualitative and quantitative designs.
				4) Many studies used single informant measures to measure family-level data	
					3) There is a need for more within-in family measurements that utilize multi-level and multi-measurement approaches.
				5) Many studies used single item measures.	
				6) Many studies adjusted for gender, SES and ethnicity as covariates, but left out other influential covariates such as maternal BMI and parental perception of child/adolescent weight.	4) There is a need to use systemic outcome variables. More family system variables should be studies.
					5) Examine possible mediator or moderator effects of the family domains.
					6) Important to include covariates when studying familial correlates of child/adolescent obesity.
Rasmussen et al., 2006 [27]	Fruit and/or vegetable intake	Socio-demographic factors, personal factors, family-related, friends-related factors, school-related factors, meal patterns, TV watching, eating fast food.	The determinants supported by the greatest amount of evidence are social-economic position, preferences, parental intake, and home availability/accessibility. For nutritional knowledge, self-efficacy and shared family meals the evidence for positive associations is rather convincing.	1) Publications may have been missed due to the search strategy.	1) More studies on the influence of the family setting for influencing fruit and vegetable intake among children and adolescents are needed to enable health promoters to make evidence based decisions.
				2) Within this review only significant associations are considered.	
					2) Observational studies analyzing fruit and vegetable intake in a school setting are still lacking.
				3) Many papers include analyses based on small study samples and samples that are non-representative or only representative of a restricted geographical area.	
					3) Future international comparative surveys should enable investigations of national level factors of importance e.g. price levels, policy, guidelines, supply, and exposure to mass media and commercials.
				4) Often the validity of the applied instruments are only considered very superficially or not mentioned at all.	
					4) Future research should study the influence of e.g. local access to fruit and vegetables through grocery stores, local food policies, exposure to mass media and commercials, and fruit and vegetable availability in leisure time facilities for children and adolescents, like for instance local sport clubs.
				5) There is insufficient confounder control.	
				6) Large variety of approaches for conceptualizing, operationalizing, measuring and coding the outcome variable(s) exist.	
					5) Future research would benefit from improvements in design and methodology.
					6) More longitudinal studies of children and adolescents’ fruit and vegetable intake are needed.
					7) Lack of knowledge about predictors of FVI among children and adolescents from non-western parts of the world.
					8) Future studies should keep a very broad and comprehensive theoretical scope, in order not to exclude important etiological components of importance for child and adolescent FVI.

*Note*: The overall results/limitations/recommendations of the reviews that are reported are those reported by the review authors themselves.

### Determinant-behavior relationship: correlate and outcome measures

Potential determinants of a range of dietary behavior outcomes among youth were explored, and many studies included multiple dietary behavior outcomes.

Thirteen reviews explored associations between environmental factors and dietary behavior [16,18,20,21,23-31]. Within the environmental determinants, the social-cultural environment was most often studied (n = 12) [16,18,20,21,23-30]. Thereafter, the physical environmental determinants (n = 9) [16,21,23-26,29-31], the economic/financial environmental determinants (n = 4) [16,21,29,30], and the political environment (n = 1) [27]. Three reviews explored the associations between social-cognitive determinants and dietary behavior [15,24,27]. These social-cognitive determinants included attitude, self-efficacy/perceived behavioral control, and intention in the study of McClain et al. [24] and Rasmussen et al. [27], subjective norm in the study of Rasmussen et al. [27], and self-regulation in the study of Adriaanse et al. [15]. Two of these reviews also examined the influence between sensory determinants and dietary behavior [24,27]. One review addressed the relation between habit strength and dietary behavior [17]. And finally, three reviews looked at sedentary behavior in relation to dietary behavior [19,22,27].

In total, four reviews solely explored associations between determinants and fruit and/or vegetable consumption: self-regulation [15]; physical, social-cultural and economic environmental determinants [16]; physical and social-cultural determinants [26]; and social-cultural, political, and social-cognitive determinants, sensory processes, and sedentary behavior [27]. Additionally, one review solely explored associations between habit strength and sugar-sweetened beverage intake [17], and one review solely explored association between physical and social-cultural environmental determinants and breakfast consumption [25]. The other 11 reviews explored determinants of a variety of healthful and unhealthful dietary behaviors (e.g. snacks, fruit and vegetables, soft drinks, milk, breakfast) [18-24,28-31]. Dietary behaviors most often included as outcomes in the included reviews were fruit and/or vegetable consumption (n = 14) [15,16,18-24,27-29,31], followed by sugar-sweetened beverage consumption (n = 10) [17,18,20-24,28-30], snack consumption (n = 9) [18,19,21-24,28,29,31] and breakfast consumption (n = 7) [18,20,22,23,25,28,30] (see Table 5).

### The importance and strength of evidence of potential determinants

With regard to the importance of a determinant and its strength of evidence (Table 6), most determinant-behavior relationships were coded with a zero, indicating that the findings are mixed. The following categories of determinants were found to be significantly related to dietary behavior and/or reported a (non)-significant effect size larger than 0.30 in all identified eligible studies of the included reviews assessing these categories of determinants (++ in Table 3): some aspects of social-cognitive determinants (such as attitude, self-regulation, intention and self-efficacy) and dietary behavior [15,24,27]; habit strength and sugar-sweetened beverage intake [17]; sensory processes and snacking [24]; and sedentary behavior and sugar-sweetened beverage and breakfast consumption [22]. The following categories of determinants were found to be significantly related to dietary behavior and/or reported a (non)-significant effect size larger than 0.30 in more than 75% of the identified reviews assessing these categories of determinants (+ in Table 3): the physical environment and fruit intake [26]; the social-cultural environment and fruit and vegetable intake [16,18,21,24,27-29] and sugar-sweetened beverage consumption [18,20,21,23,24,28-30]; intention, sensory processes, and knowledge for fruit and vegetable intake [24,27]; and sedentary behavior and fruit intake [22], fruit and vegetable intake and snack intake [19,22]. The evidence is mostly limited (limited, suggestive: Ls), predominantly due to the abundance of studies with cross-sectional designs so that causal or predictive relations could not be established. Systematic review on the influence of political environments, self-regulation, subjective norm and automaticity were mostly lacking in the included reviews.

## Discussion

### Main results

The multitude of studies conducted on determinants of dietary behavior among youth provides mixed and sometimes quite convincing evidence regarding associations between potential determinants and a range of dietary behaviors. However, because of the general use of cross-sectional designs in the studies covered in the available reviews, the evidence for true determinants is suggestive at best.

In particular, environmental determinants (mainly the social-cultural environment) and social-cognitive determinants have been studied quite extensively for their association with different dietary behaviors, with somewhat mixed results. The included reviews suggest that in the past decade, environmental determinants have been studied most extensively. This is an important finding in itself, suggesting a paradigm shift in the field, i.e. from a focus on social-cognitive determinants to environmental factors. This shift towards more consideration of the social-ecological approach was also seen in our umbrella review on determinants of dietary behavior in adults [14]. Other potential determinants of dietary behavior, such as automaticity, self-regulation, and subjective norm, have been studied in relatively few studies, but study results are promising. With regard to the outcomes investigated, most reviews explored relations of potential determinants with fruit and/or vegetable intake.

In the reviewed papers we found evidence that the social-cultural environment, such as the familial influence (e.g. [21,24,27,28]) is a significant correlate of fruit and vegetable intake and snack consumption in youth in more than 75% of the available studies (see Table 7). Parents, as gatekeepers of the home food supply, can influence children’s eating behavior either through the use of specific food parenting practices (i.e. context-specific acts of parenting on child eating including encouraging of food variety and controlling a child’s intake of unhealthy products) or through the indirect influence of general parenting [28]. Social-cognitive determinants have been studied often, but the evidence regarding their importance is limited (i.e. suggestive at best). Intention, a proximal indicator of actual behavior, was found to be a significant determinant of fruit and vegetable intake, snack intake, and sugar-sweetened beverage intake [24,27]. Socio-cognitive theories such as the Theory of Planned Behavior [6] are indicated to have limited value in predicting the translation of intention into action. This limitation is addressed in reviews on the constructs of habit [17] and implementation intentions [15]. The review on implementation intentions showed considerable support for the effect of implementation intentions on increasing fruit and vegetable intake (medium effect size) among youth. However, the effect of implementation intentions on the reduction of unhealthy eating patterns was less convincing. Habit strength was one of the factors to be significantly related to sugar-sweetened beverage intake with moderate to strong effect sizes in all identified eligible studies of the review of Gardner et al. [17]. Automatic processes, including habit strength appears to reduce the utility of cognitive factors for the prediction or association with dietary behavior [17]. Additionally, screen time was found to be consistently associated with dietary behavior [19,22,27]. The included reviews provide evidence that the amount of screen time was significantly related to dietary behavior; screen time was positively associated with snack and sugar-sweetened beverage intake [19-22] and inversely associated with fruit and vegetable intake [19-27]. An important mechanism linking screen time to unhealthy dietary behavior is exposure to marketing of unhealthy foods and beverages through screens [35,36]. The food and beverages depicted in these advertisements are predominantly unhealthy foods high in fat, salt and sugar [35,37]. Sedentary activities and unhealthy dietary behavior have repeatedly been found to cluster [38-40] and may also share similar environmental cues causing these behaviors to co-occur. Sedentary behavior offers a context for the consumption of energy-dense food products, disrupting the habituation to food cues.

**Table 7:** **Summary of the results from reviews about determinants of dietary behavior among youth: Importance of a determinant and strength of evidence**

	**Dietary behavior**					
**Determinants**	**Fruit**	**Vegetable**	**Fruit & vegetable**	**Snack/fast food**	**Sugar-sweetened beverage**	**Breakfast**
Physical environment	+, Ls [26]	0, Ls [26]	0, Ls [15,21,24,29,31]	0, Ls [21,23,24,29,31]	0, Ls [21,23,24,29,30]	0, Ls [23,25]
Social-cultural environment	0, Ls [20,23,26-28]	0, Ls [20,23,26-28]	+, Ls [16,18,21,24,27-29]	0, Ls [18,21,23,24,28,29]	+, Ls [18,20,21,23,24,28-30]	0, Ls [18,20,23,25,28,30]
Economic/financial environment			0, Ls [16,21,29]	0, Ls [21,29]	-, Ls [21,29,30]	-, Lnc [30]
Political environment			0, Ls [27]			
Attitude	0, Lnc [24]	0, Lnc [24]	0, Ls [24,27]	++, Ls [24]	++, Lnc [24]	
Subjective norm			++, Lnc [27]			
Self-efficacy/perceived behavioral control	0, Ls [24]	0, Ls [24]	0, Ls [24,27]	0, Ls [24]	++, Lnc [24]	
Intention	++, Ls [24]	++, Lnc [24]	+, Ls [24,27]	++, Ls [24]	++, Lnc [24]	
Self-regulation			++, Ls [15] (implementation intentions)			
Habitual behavior, automaticity					++, Lnc [17]	
Sensory perceptions, perceived palatability foods			+, Ls [24,27]	++, Lnc [24]	--, Lnc [24]	
Other, knowledge	0, Ls [24]	0, Ls [24]	+, Ls [24,27]	0, Ls [24]	++, Lnc [24]	
Other, sedentary behavior	+, Ls [19,22,27]	0, Ls [19,22,27]	+, Ls [19,22,27]	+, Ls [19,22]	++, Ls [22]	++, Ls [22]

*Note*: Importance of a determinant: ++, +, 0, −, −- (see Table 3); strength of evidence (see Table 4): Co (Convincing evidence), Pr (Probable evidence), Ls (Limited, suggestive evidence), Lnc (Limited, no conclusion); studies including determinants such as stress and risks and dietary behavior such as milk and meat intake not included in this table.

Systematic reviews on the influence of political environments, self-regulation, subjective norm and habitual behavior were mostly lacking in the included reviews. In addition, some types or categories of potential determinants were not covered in the present umbrella review because we did not come across systematic reviews of such determinants. For instance, although we found two systematic reviews on sensory determinants of dietary behavior [24,27], most of the reviews were excluded as they did not comply to the quality standards of systematic reviews that we used as an inclusion criteria, e.g. [41]. This does not necessarily imply that such factors as taste and preferences are not important, but just that these have not been covered well at present in systematic reviews. Furthermore, it should be noted that lack of evidence for the importance of a possible determinant is not the same as evidence that the determinant is not important; since lack of well-designed studies is often the main reason for lack of evidence. We need to try to distinguish between well-researched determinants and still no evidence for importance, and determinants that have just not been studied (well enough) to make meaningful conclusions.

### Limitations and methodological issues

Several limitations should be taken into consideration in reviewing these findings. These include the cross-sectional nature of many studies relying on self-report measures; heterogeneity of conceptualization, measurement, samples and analyses used, making it difficult to compare results between studies; inability to conduct a meta-analysis; lack of validity and reliability of dietary intake and correlate measures; and categorization of determinants into more global categories thereby losing important information. Additionally, the systematic reviews included a wide age range, i.e. respondents from birth to 18 years. During childhood many developmental transitions take place that may imply differential importance of distinct behavioral determinants. For instance, parents are highly responsible as gatekeepers of the home food supply for their children’s dietary intake behavior. However, parental influence decreases with advancing age of the child as the child is increasingly exposed to other environments (e.g. school environment, peer influences).

In addition to the quality of the research design, the fact that some determinants have not been extensively studied yet, studies of some types of determinants have not been reviewed systematically, and the lack of robust results from this umbrella review may also be explained by the fact that groups or types of determinants are often studied in relative isolation. For instance, studies in which different categories of determinants - e.g. sensory determinants, self-regulation, and political environmental factors - were studied with integrative approaches are largely lacking. Such studies would allow for exploration and testing of mediating and moderating pathways between these determinants in influencing dietary behavior. Already some studies combining environmental and social-cognitive determinants have been reported in recent years and do support such mediating and moderating pathways, e.g. [42-45].

This is the first umbrella review that provides an overview of reviewed research regarding a broad range of potential determinants of dietary behavior in youth. Umbrella reviews in itself are, however, also prone to bias in various ways. Differences in reviewing methodology and reporting were apparent, as well as differences in for example categorizations of the determinants. By nature, umbrella reviews lead to loss of detail. In addition, some individual studies are included in multiple reviews which may have led to an overrepresentation of single studies in our results. Finally, we excluded reviews that primarily addressed biological determinants or papers with summative outcomes such as caloric intake, and we also did not include reviews that focused on qualitative data.

## Conclusions and recommendations

The evidence gathered in our umbrella review suggests that intention and sedentary behavior have the strongest evidence base as determinants of healthy and unhealthy dietary behavior in youth. The influence of distinct determinants may, however, be stronger in interaction with other influences. We would advocate for studies that address combined, mediating and interactive influences on dietary behavior [46]. Such studies are advocated to include behaviors that have been found to cluster with dietary behavior, such as sedentary behavior. Other recommendations include the need for better designed studies, beyond mere cross-sectional research, −i.e. more longitudinal and experimental or intervention research, and research using natural experiments-, larger samples among specific age groups, and more valid and reliable measures (dietary behavior and correlates). Our results underline the importance of embracing theories and factors additional to determinants derived from socio-cognitive theories that are often used to inform interventions to promote healthy dietary behaviors. Theories that are promising of further research for determinants of dietary behavior research include habit theory and (social-) ecological models of health behavior.
